# Acceptability and perceived feasibility of restroom motion sensors for overdose detection and response in public bathrooms: A cross-sectional study of businesses and community organizations

**DOI:** 10.1016/j.drugalcdep.2025.112788

**Published:** 2025-07-09

**Authors:** Joseph G. Rosen, Ryan Koch, Rehan Aslam, Gabrielle Riendeau, Maxwell S. Krieger, Michelle McKenzie, Susan E. Ramsey, Traci C. Green, Josiah D. Rich, Ju Nyeong Park

**Affiliations:** a Division of General Internal Medicine, Rhode Island Hospital, Providence, RI, United States; b Department of Medicine, The Warren Alpert Medical School, Brown University, Providence, RI, United States; c Department of Epidemiology, School of Public Health, Brown University, Providence, RI, United States; d Department of Psychiatry and Human Behavior, The Warren Alpert Medical School, Brown University, Providence, RI, United States; e Department of Emergency Medicine, The Warren Alpert Medical School, Brown University, Providence, RI, United States; f Opioid Policy Research Collaborative, The Heller School for Social Policy and Management, Brandeis University, Waltham, Massachusetts, United States; g Division of Infectious Diseases, The Miriam Hospital, Providence, RI, United States

**Keywords:** Overdose detection technologies, Solitary drug use, Opioids, Naloxone, United States

## Abstract

**Purpose:**

Solitary drug use amplifies fatal overdose risk for people who use drugs. Restroom motion sensors (RMS) are emerging technologies to enhance detection and facilitate rapid response to overdoses in high-traffic public restrooms (where unmonitored drug use is likely to occur), but there has been limited study of employee and staff perspectives on the perceived value and appropriateness of RMS for their workplaces.

**Methods:**

From November 2022 to April 2024, we identified business, health, and community organizations in opioid overdose hotspots across eight townships in Rhode Island (United States of America), surveying managers and shift supervisors about the acceptability and perceived feasibility of RMS. We analyzed data descriptively, identifying bivariate correlates of RMS acceptability and feasibility, respectively, using Fisher’s exact tests of association.

**Results:**

Participants (median age: 35 years, 53 % cisgender women) across 100 workplaces exhibited low awareness (4 %) but high comfort with RMS being installed (73 %) and confidence (66 %) in implementing RMS in their workplaces in the future. Organizations without staff adequately trained in overdose response were more likely than organizations with trained staff (81 % vs. 64 %, *p* = 0.055) to endorse confidence in RMS implementation.

**Conclusions:**

Management and shift supervisors in heterogenous occupational contexts endorsed RMS’ compatibility and utility in their workplaces, despite low prior awareness of the technology. Efforts to bolster staff competencies in overdose response and equipping workplaces with the necessary tools (i.e., onsite naloxone) to respond appropriately to onsite overdoses will be imperative to successful implementation of RMS in businesses and community organizations.

## 1. Introduction

Over one million American lives have been lost to drug overdose since 2000—driven, in part, by cascading policy failures (i.e., mass incarceration, opioid prescribing, access inequities to drug treatment and harm reduction services) that amplify overdose risk (Spencer et al., 2022). More recently, the emergence of illicitly manufactured fentanyl and other potent contaminants in the unregulated drug supply have exacerbated the U.S. overdose crisis. Over half of U.S. overdose decedents are discovered alone at the time of death—suggesting that the expected benefits of community naloxone distribution and saturation, as well as new naloxone access laws/policies, are being attenuated in the context of unmonitored, solitary drug use (Mattson et al., 2018). High-traffic restrooms in workplaces are settings where staff or members of the public may use drugs privately, and subsequently, experience an unmonitored, potentially fatal overdose (Wolfson-Stofko et al., 2017; Buchheit et al., 2021; Gagnon et al., 2023). As novel synthetic agents (i.e., fentanyl analogs, nitazines, xylazine, medetomidine) continue flooding the unregulated drug market, additional strategies are needed to facilitate timely overdose detection and response in public settings.

Overdose detection technologies (ODTs), including end-user-activated and automated detection systems designed specifically for people who use drugs alone, are emerging tools that can notify nearby responders of people experiencing a potential overdose (Lombardi et al., 2023; Park et al., 2023; Rioux et al., 2023). Wearable, mobile, and fixed-location ODTs are in various stages of development and implementation; restroom motion sensors (RMS) are ODTs designed to detect the absence of micromovements in single- or multi-stall restrooms and transmit private alerts to designated responders or activate nearby alarms (Lombardi et al., 2023; Park et al., 2023). Two RMS products, the Brave Sensor (Brave Technology Co-op, Vancouver, British Columbia, Canada) and the Life Saver Bathroom System (Life Saver Alert LLC, Milford, New Hampshire, United States of America), have been introduced in housing, clinical, and other service delivery contexts across North America (Buchheit et al., 2021; Fozouni et al., 2020; Welwean et al., 2023).

Recent scholarship has demonstrated RMS’ effectiveness in detecting and facilitating timely responses to suspected overdoses across occupational contexts, with one North American study documenting one RMS product’s detection of eight overdoses across 148 installations (Welwean et al., 2023). However, only two studies have assessed staff perspectives of RMS installation and implementation—both of which were exclusively qualitative in nature and limited to clinical care settings (Buchheit et al., 2021; Rioux et al., 2024). Because employees are most likely to discover an overdose in workplace restrooms (Wolfson-Stofko et al., 2017), assessments of RMS compatibility and utility in diverse workplaces are critical to guiding future scale-up efforts. Accordingly, our cross-sectional study characterized employee perspectives of RMS installation and implementation in heterogeneous occupational settings, including commercial and retail establishments, community organizations, and clinical facilities in Rhode Island—a U.S. state with high burdens of fatal and non-fatal opioid-involved overdoses (King et al., 2022; Omari et al., 2024; Weidele, Hallowell, 2024).

## 2. Methods

We began by mapping non-fatal opioid overdose hotspots in Rhode Island using timestamped emergency medical services (EMS) responses to non-fatal opioid-involved overdoses from 2020 to 2022 (*N* = 5377), captured and collated by the Rhode Island Department of Health via the Rhode Island Emergency Medical Services Information System (RIEMSIS) ImageTrend (Rodriguez et al., 2024). Census block groups with per-capita non-fatal overdose rates in the top 10–20% across all geographies were classified as hotspots (*N* = 107), which were further stratified by non-outdoor overdose location (housing [*n* = 69] versus non-housing [*n* = 38]).

We then used opensource web-mapping platforms to identify and locate food service, gas stations, retail establishments, bars/nightclubs, hotels/motels, housing providers, healthcare facilities, harm reduction drop-in centers, and other public service organizations within mapped overdose hotspots—spanning eight townships across the state. From November 2022 to April 2024, we approached organizations in-person or via telephone, explaining the purpose of the study and screening eligible representatives (i.e., aged 18+ years, worked as a manager or shift supervisor at an organization with a publicly accessible restroom for 6+ months, English language comprehension) from identified workplaces for survey participation.

After providing verbal informed consent, participants completed a structured, interviewer-administered questionnaire (averaging 15–20min) on handheld tablet computers. Surveys elicited occupational experiences with onsite medical emergencies, including overdoses; measures and protocols established for overdose detection and response (e.g., establishing standard operating procedures, training staff in overdose response, having naloxone available onsite); and RMS awareness (“Have you heard of any of the following ODTs?”), feasibility (“I am *confident* that this building can use RMS for detecting and responding to overdoses”), acceptability (“I feel *comfortable* with having RMS installed for detecting and responding to overdoses in this building”), willingness to have employees/staff trained in RMS prior to installation (“strongly disagree” to “strongly agree”), and willingness to participate in a future RMS pilot study (*yes* versus *no/unsure*). Only one survey was administered per workplace. Participants received $20 cash for survey completion.

Data were managed and analyzed in Stata/SE 15.1 (StataCorp LLC, College Station, Texas, United States of America). After calculating descriptive sample statistics (frequencies and proportions), dichotomized measures of RMS feasibility and acceptability (agree versus unsure/disagree) were derived from 5-point Likert scales (“strongly disagree” to “strongly agree”). Binary measures of perceived RMS feasibility and acceptability, respectively, were then compared across organization types (*commercial establishments* versus *communal housing facilities* versus *social service organizations*) and workplace characteristics using Fisher’s exact tests of association. Given the paucity of missing data (3%), complete case analysis was implemented across descriptive and bivariate analyses. To account for the small survey sample size, thresholds for promising, albeit non-statistically significant, differences were set *a priori* at the *p* < 0.2 level.

### 2.1. Ethics statement

All individuals provided verbal informed consent prior to study enrollment and participation. The study protocol was reviewed and approved by the Brown University Health Institutional Review Board (#1886083, #1911766).

## 3. Results

Of the 280 workplaces identified and mapped within overdose hotspots, 41 (14.7%) were unable to be reached; 37 (13.2%) were deemed ineligible due to the absence of publicly accessible restrooms; and 102 (36.4%) declined participation or did not respond after expressing initial interest in participating. Managers or shift supervisors from one hundred (*N* = 100) workplaces completed the survey (participation rate: 35.7%), the largest proportions of participants were from food service (27%) and retail establishments (16%). Nearly half (48%) of respondents were aged 18–34 years (median: 35 years, interquartile range: 30–44 years), with 53% identifying as cisgender women and 70% as non-Hispanic White. Fewer than half reported having any measures or protocols in place for overdose detection (35%), staff trained in overdose response (48%), and naloxone available onsite for opioid overdose reversal (41%). Over one-third of organizations reported a history of an onsite overdose (37%), and most reported experiences with other, non-overdose-related onsite medical emergencies (71%).

While only 4% of surveyed employees had any familiarity with RMS, two-thirds (66%) expressed confidence in the implementation of RMS at their respective organization, and nearly three-fourths (73%) expressed comfort with the hypothetical installation and use of RMS in their workplaces. Nearly two-thirds (59%) of participants indicated willingness to have employees or other staff receive requisite training in RMS prior to installation, and 57% expressed interest in participating in a future RMS pilot study.

**Fig. 1.**
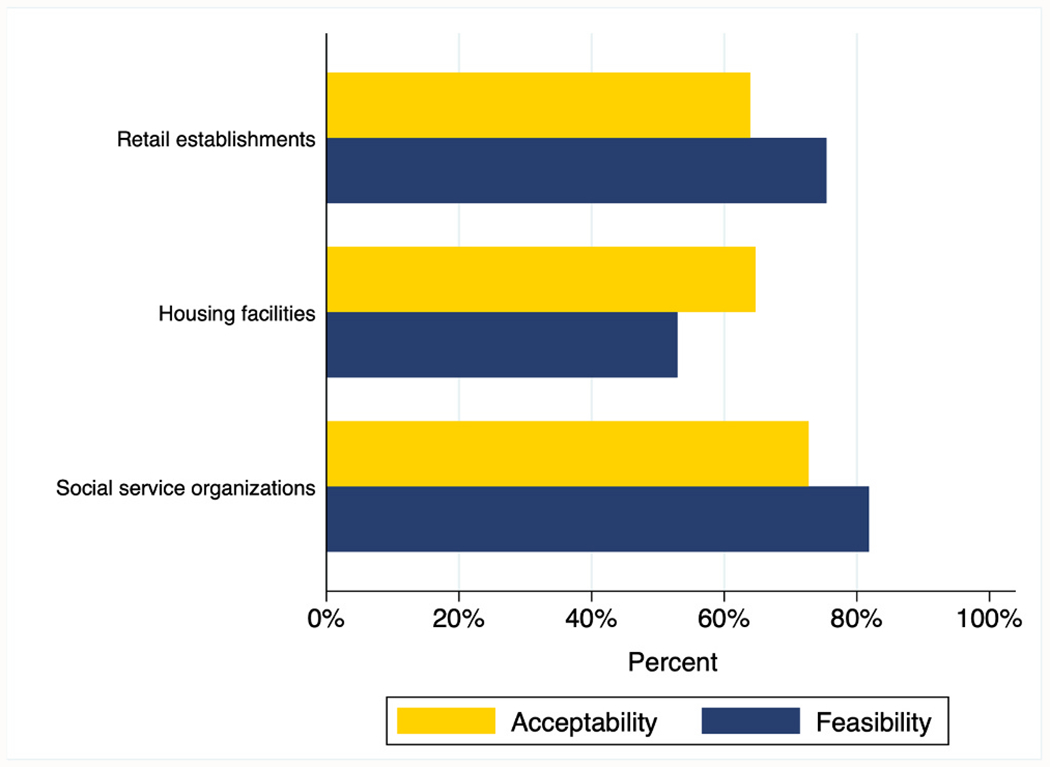
Perceived feasibility and acceptability of restroom motion sensors, by organization type (N = 100).

Table 1 presents endorsed feasibility and acceptability, respectively, of RMS by workplace characteristics. Organizations with staff who were untrained in overdose response were more likely to endorse confidence in RMS capacity to detect and facilitate response to onsite overdoses, compared to those with previous training (81 % vs. 64 %, *p* = 0.055). Likewise, retail establishments (75 %) and social service organizations (82 %) were more likely than commercial or communal housing providers (53 %) to express confidence in RMS’ capacity to detect and facilitate response to onsite overdoses (*p* = 0.132) (see Fig. 1). No meaningful differences in workplace characteristics were identified between organizations expressing comfort and discomfort, respectively, with RMS installation onsite.

## 4. Discussion

Surveys of managers and shift supervisors at workplaces located in overdose hotspots across Rhode Island revealed high perceived feasibility and acceptability of RMS to detect and facilitate response to onsite overdoses in publicly accessible restrooms. Workplaces where staff had limited capacity or training in overdose response were most likely to perceive RMS as feasible solutions to restroom overdose detection and response. Because these technologies facilitate automatic, timely detection of potential restroom overdoses, they could circumvent onerous requirements on workplaces to implement manual restroom checks or other forms of surveillance that may potentially implicate an untrained workforce in traumatic overdose response activities (Buchheit et al., 2021). RMS, among other ODTs, have great potential to increase layperson capacity for overdose response while making publicly accessible restrooms safer environments for people who use drugs and employees/staff alike.

Despite high enthusiasm for RMS among surveyed managers and shift supervisors, these technologies may not be appropriate for all workplaces—as exemplified by setting-specific variations in perceived RMS feasibility. Specifically, commercial (hotels/motels) or supportive housing providers (i.e., housing shelters), relative to retail establishments and social service organizations, were less likely to perceive RMS as feasible. These differences may be artifacts of overdose characteristics and dynamics at these sites, with many housing establishments—especially staff at hotels/motels and single-room occupancy housing—likely perceiving drug use to occur in the privacy of individual bedrooms and restrooms rather than publicly-accessible restrooms (Rowe et al., 2019). Push-activated, wall-mounted technologies requiring end-user activation (i.e., Brave Button) or remote overdose monitoring services (i.e., mobile applications and hotlines) may, thus, be better suited for people using drugs alone in private housing contexts (Lombardi et al., 2023; Rioux et al., 2023).

The present study did not explicitly capture reasons underpinning perceived (dis)comfort and (lack of) confidence in RMS installation and implementation; was implemented exclusively with English-speaking individuals; excluded participation from frontline (non-managerial) staff; and is susceptible to recall/response biases. Nevertheless, findings underscore the high burdens of overdoses occurring in heterogeneous workplaces, but suboptimal training and resources for employees and staff to respond appropriately and adequately to onsite overdoses. RMS installation in these occupational contexts may be an important vehicle to increasing employee/staff competencies in overdose response and infusing overdose response resources (i.e., naloxone distribution and possession) into impacted workplaces. If RMS are proven to be accurate, safe, and effective, efforts to disseminate information about these technologies, including their relative affordability could help address the low awareness of RMS found among workplaces. To appropriately guide implementation and scale-up of these technologies, future research should interrogate perceptions of RMS, including acceptability, among people who use drugs, their families, and other potential responders to overdose emergencies.

## Figures and Tables

**Table 1:** Perceived feasibility and acceptability of restroom motion sensors (RMS), by workplace characteristics (*N* = 100).

Workplace characteristics (*n*, %)	Feasibility: “I am confident that this building can use RMS for detecting and responding to overdoses”			Acceptability: “I feel comfortable with the installation of RMS for detecting and responding to overdoses”		
	“*Strongly Disagree*” to “*Unsure*”	“*Agree*” to “*Strongly Agree*”	*p*-value	“*Strongly Disagree*” to “*Unsure*”	“*Agree*” to “*Strongly Agree*”	*p*-value
Locality			0.549			0.435
Urban	20 (27.4)	53 (72.6)		24 (32.9)	49 (67.1)	
Suburban	7 (25.9)	20 (74.1)		10 (37.0)	17 (63.0)	
Organization type			0.132			0.796
Retail establishments	15 (24.6)	46 (75.4)		22 (36.1)	39 (63.9)	
Commercial or communal housing	8 (47.1)	9 (52.9)		6 (35.3)	11 (64.7)	
Social service organizations	4 (18.2)	18 (81.8)		6 (27.3)	16 (72.7)	
Naloxone availability onsite			0.400			0.269
No	17 (28.8)	42 (71.2)		22 (37.3)	37 (62.7)	
Yes	10 (24.4)	31 (75.6)		12 (29.3)	29 (70.7)	
Staff trained in naloxone response			0.055			0.221
None or unsure	10 (19.2)	42 (80.8)		20 (38.5)	32 (61.5)	
Some or all	17 (35.4)	31 (64.6)		14 (29.2)	34 (70.8)	
Measures/protocols for OD prevention			0.489			0.571
None or unsure	17 (27.0)	46 (73.0)		22 (34.9)	41 (65.1)	
Any	10 (29.4)	24 (70.6)		12 (35.3)	22 (64.7)	
Manual bathroom checks for OD detection			0.322			0.219
No or unsure	5 (21.7)	18 (78.3)		6 (26.1)	17 (73.9)	
Yes	22 (29.7)	52 (70.3)		28 (37.8)	46 (62.2)	
History of onsite OD			0.403			0.320
Never or unsure	16 (25.4)	47 (74.6)		23 (36.5)	40 (63.5)	
Ever	11 (29.7)	26 (70.3)		11 (29.7)	26 (70.3)	
History of onsite medical emergency			0.365			0.222
Never or unsure	9 (31.0)	20 (69.0)		12 (41.4)	17 (58.6)	
Ever	18 (25.4)	53 (74.6)		22 (31.0)	49 (69.0)	

*Notes*: OD = overdose
